# Supplementary material for: Hybrid Models and Biological Model Reduction with PyDSTool
Source: PLoS Comput Biol. 2012 Aug 9;8(8):e1002628. doi: 10.1371/journal.pcbi.1002628 (PMC3415397; doi:10.1371/journal.pcbi.1002628)
Supplement: Text S4 — Complete source code for the PyDSTool package (version 0.88.120504). Includes API documentation and help files linking to web pages. This file is identical to the current public release on Sourceforge.net. (ZIP) [file pcbi.1002628.s004.zip › PyDSTool/html/PyDSTool.Toolbox.optimizers.criterion.facilities-pysrc.html]

xml version="1.0" encoding="ascii"?


PyDSTool.Toolbox.optimizers.criterion.facilities


| Home | Trees | Indices | Help | PyDSTool |
| --- | --- | --- | --- | --- |

Package PyDSTool ::
Package Toolbox ::
Package optimizers ::
Package criterion ::
Module facilities

[hide private]

[frames] | no frames]

# Source Code for Module PyDSTool.Toolbox.optimizers.criterion.facilities

```
 1  
 
 2  # Matthieu Brucher
 
 3  # Last Change : 2007-08-24 10:05
 
 4  
 
 5  """
 
 6  Proposes a way to create a composite criterion
 
 7  """ 
 8  
 
 9  __all__ = ['criterion'] 
10  
 
11  from criteria import IterationCriterion, RelativeValueCriterion, RelativeParametersCriterion, GradientCriterion 
12  from composite_criteria import OrComposition 
13  
 


14 -def criterion(**kwargs):


15    """
 
16    Creates a composite criterion based on the formal parameters :
 
17      - iterations_max indicates the maximum number of iteration
 
18      - ftol is the maximum relative change of the value function
 
19      - xtol is the maximum relative change of the parameters
 
20      - gtol is the maximum gradient
 
21    """ 
22    l = [] 
23    if 'iterations_max' in kwargs: 
24      l.append(IterationCriterion(kwargs['iterations_max'])) 
25    if 'ftol' in kwargs: 
26      l.append(RelativeValueCriterion(kwargs['ftol'])) 
27    if 'xtol' in kwargs: 
28      l.append(RelativeParametersCriterion(kwargs['xtol'])) 
29    if 'gtol' in kwargs: 
30      l.append(GradientCriterion(kwargs['gtol'])) 
31  
 
32    return OrComposition(*l)

33
```

  


| Home | Trees | Indices | Help | PyDSTool |
| --- | --- | --- | --- | --- |

|  |  |
| --- | --- |
| Generated by Epydoc 3.0.1 on Fri May 4 15:24:18 2012 | http://epydoc.sourceforge.net |
